# Isolation and identification of indigenous plant growth promoting rhizobacteria from Himalayan region of Kashmir and their effect on improving growth and nutrient contents of maize (*Zea mays* L.)

**DOI:** 10.3389/fmicb.2015.00207

**Published:** 2015-03-17

**Authors:** Mahwish Zahid, M. Kaleem Abbasi, Sohail Hameed, Nasir Rahim

**Affiliations:** ^1^Department of Soil and Environmental Sciences, Faculty of Agriculture, The University of PoonchRawalakot, Azad Jammu and Kashmir, Pakistan; ^2^Technical Services Division, Nuclear Institute of Biotechnology and Genetic EngineeringFaisalabad, Pakistan

**Keywords:** biofertilizer, growth attributes, isolation, PGPR, rhizobacteria, 16S rRNA gene sequencing

## Abstract

Introduction and exploitation of plant growth promoting rhizobacteria (PGPR) in agro-ecosystems enhance plant–microbes interactions that may affect ecosystems sustainability, agricultural productivity, and environmental quality. The present study was conducted to isolate and identify PGPRs associated with maize (*Zea mays* L.) from twenty sites of Himalayan region of Hajira-Rawalakot, Azad Jammu and Kashmir (AJK), Pakistan. A total of 100 isolates were isolated from these sites, out of which eight (HJR_1_, HJR_2_, HJR_3_, HJR_4_, HJR_5_, MR_6_, HJR_7_, HJR_8_) were selected *in vitro* for their plant growth promoting ability (PGPA) including phosphorus solubilization, indole-3-acetic acid (IAA) production and N_2_ fixation. The 16S rRNA gene sequencing technique was used for molecular identity and authentication. Isolates were then further tested for their effects on growth and nutrient contents of maize (*Z. mays* L.) under pouch and pot conditions. The *16S rRNA* gene sequencing and phylogenetic analysis identified these isolates belong to *Pseudomonas* and *Bacillus* genera. The isolates promoted plant growth by solubilizing soil P which ranged between 19.2 and 35.6 μg mL^-1^. The isolates HJR_1_, HJR_2_, HJR_3_, and HJR_5_ showed positive activity in acetylene reduction assay showing their N_2_-fixation potential. All eight isolates showed the potential to produce IAA in the range of 0.9–5.39 μg mL^-1^ and promote plant growth. Results from a subsequent pot experiment indicated PGPRs distinctly increased maize shoot and root length, shoot and root dry weight, root surface area, leaf surface area, shoot and root N and P contents. Among the eight isolates, HR_3_ showed a marked P-solubilizing activity, plant growth-promoting attributes, and the potential to be developed as a biofertilizers for integrated nutrient management strategies.

## Introduction

Intensive farming practices that achieve high yield require continuous application of chemical fertilizers in our agro-ecosystems. However, the prices and availability of these chemical fertilizers become the limiting factor for crop production especially in developing countries around the world. Continuous application of N fertilizers may result in negative impacts on agro-ecosystem such as leaching, pollution of water resources, gaseous emissions to atmosphere thus causing irreparable damage to the overall ecosystem and environment. Similarly, phosphorus (P) is one of the major essential macronutrients for biological growth and application of P fertilizers is indispensable component for crop production. However, the availability of P to plants is a serious issue because of its fixation and precipitation behavior in soil which lowers the efficiency of added P. It has been reported that more than 80% of applied P as fertilizers precipitates in the presence of metal ion complexes in soil (Ca^2+^ in calcareous soils and Fe^3+^ and Al^3+^ in acidic soils; Qureshi et al., 2012). In addition to these constraints, the prices of P fertilizers jumped up several folds during recent years making P fertilizers not-affordable to a common resource poor farmer.

Introduction of plant growth promoting rhizobacteria (PGPR) including phosphate solubilizing bacteria (PSB) as biofertilizers is suggested as a sustainable option for the improvement of nutrient availability, plant growth, and yields (Vessey, 2003). Use of microbial consortia in the form of biofertilizers for reducing the use of chemical fertilizers without compromising yield is presently an important feature of research in the field of agriculture, microbiology, and biotechnology (Minorsky, 2008). The search for diverse PGPRs is gaining serious attention and efforts are made to exploit them as biofertilizers for various economically important crops.

During the last two decades use of microbial techniques and introduction of rhizobacteria in agriculture has increased tremendously due to their potential for N_2_-fixation and P solubilization thus increasing N and P uptake by the plants and therefore yields (Vessey, 2003). Successful results of using PGPR species including *Azospirillum, Bacillus, Pseudomonas* and *Enterobacteria* on maize, canola, wheat, and other horticultural crops have been achieved both in the laboratory and in the field under variable ecological conditions (Abbasi et al., 2011; Almaghrabi et al., 2013; Hussain et al., 2013; El-Sayed et al., 2014; Lavakush et al., 2014; Mehta et al., 2014). However, soil–plant–microbe interactions are complex phenomena and detailed explanation had been reported in literature that shows how this interaction influences the plant health and productivity (Mehta et al., 2014). Studies have shown that PGPR strains vary widely and their growth promoting ability may be highly specific to certain plant species, cultivar, soil, and genotype (Lucy et al., 2004). Under such conditions, knowledge of native bacterial population and their identification is important for understanding their distribution and diversity.

It is important to explore and identify region specific microbial strains which can be used as potential plant growth promoters to achieve higher yields under specific ecological and environmental conditions (Fischer et al., 2007). Easy and rapid molecular techniques can be developed to perform microbial characterization. DNA sequences in 16S–23S are known to exhibit a great deal of sequences and length variation which are used to differentiate genera, species up to strain level. Sequence analysis of 16S rRNA gene is more exclusively studied and well-established method for phylogenetic and taxonomic studies (Tan et al., 2001).

Knowledge of the native bacterial population, their characterization and identification is required for understanding the distribution and diversity of indigenous bacteria (Chahboune et al., 2011). With the increasing awareness about the economic and environmental consequences of the use of chemical fertilizers, it is important to explore region specific microbial strains which can be used as a potential plant growth promoter to achieve desired results. The use of indigenous PGPR can be an added advantage since it can easily acclimatize to the natural conditions and enhance the plant–microbe interactions (Verma et al., 2013). Presently, there is very little documentation on the occurrence or utilization of PGPRs in the study region. Therefore, the objectives of this study were (i) to isolate native bacterial strains from the maize (*Zea mays* L.) rhizosphere under *in vitr*o conditions and to characterize these isolates on the basis of morphological and physiological attributes as well as by 16S rRNA sequence analysis (ii) to assess the PGPAs of these isolates *in vivo* and their effect on the nutrient contents (N and P) of maize plants at early growth stages.

## Materials and Methods

### Sample Collection, Bacterial Isolation, and Physiological Characterization

The soil used in this study was collected from twenty different maize growing sites of Hajira-Rawalakot, AJK, Pakistan. The soil in the study site was Humic Lithic Eutrudepts (Inceptisols). Soil samples were collected from 0 to 20 cm depth at random from five different locations at each site and mixed well. The field-moist soil was passed through a 4 mm sieve to eliminate coarse rock and plant material, thoroughly mixed to ensure uniformity and stored at 4^∘^C prior to use. A sub-sample of about 0.5 kg was air- dried and passed through 2-mm sieve and used for the determination of physical and chemical characteristics (**Table 1**). Soil pH was determined in distilled water with a glass electrode (soil:H_2_O ratio 1:2.5 w/v). Soil total N was determined by the Kjeldahl method (Bremner and Mulvaney, 1982). Soil organic matter was determined using a modified Mebius method (Nelson and Sommers, 1982). Available P from soil samples was determined according to Soil and Plant Analysis Laboratory Manual (Ryan et al., 2001) using AB-DTPA method modified by Soltanpour and Workman (1979). Exchangeable K was determined using a flame photometer following soil extraction with 1 N ammonium acetate (COOCH_3_NH_4_; Simard, 1993). The bacterial population was estimated by most probable numbers (PMN) count according to method described by Mirza et al. (2001).

**Table 1:** **Physico-chemical properties of soil samples collected for isolation studies from different sites of maize growing areas in Hajira, Rawalakot, Azad Jammu and Kashmir, Pakistan**.

Location/sites	MPN (cfu g^-1^)	Organic matter (%)	Total N (%)	Available P (mg kg^-1^)	pH
Upper Kharange	5.2 × 10^-6^	1.77	0.2	7.20	7.4
Thanda Gameer	6.4 × 10^-6^	1.73	0.04	8.15	7.9
Barigalli	7.0 × 10^-6^	0.38	0.23	7.81	7.8
Numble hut	1.4 × 10^-6^	1.41	0.27	8.02	7.3
Kalaran	1.8 × 10^-8^	0.40	0.15	7.68	7.2
Nazar Khawaja	7.1 × 10^-8^	1.34	0.26	13.48	7.3
Kot Koyan	4.8 × 10^-6^	0.88	0.04	9.49	7.6
Potha	1.4 × 10^-7^	1.11	0.06	9.07	7.7
Polus	2 × 10^-6^	1.24	0.07	8.45	7.4
Chatra	2.2 × 10^-8^	1.84	0.04	8.86	7.0
Manoria	1.6 × 10^-6^	1.12	0.08	10.11	8.0
Dawarandi	7.7 × 10^-8^	1.10	0.03	7.24	7.1
Shar Madarpur	6.0 × 10^-8^	0.72	0.02	7.81	7.9
Tetrinote	4.3 × 10^-6^	0.98	0.01	9.04	7.2
Kakuta	8.3 × 10^-6^	1.76	0.06	8.38	7.6
Buttle	4.1 × 10^5^	0.35	0.04	8.48	7.9
Sehra	3.48 × 1^8^	1.38	0.03	6.45	7.9
Tahi	6.6 × 10^-6^	0.34	0.04	7.07	8.1
Mandole	6.2 × 10^-8^	1.26	0.07	9.45	7.6
Hajira	8.2 × 10^-8^	1.48	0.06	8.56	8.3

*MPN, most probable number.*

The soil samples were processed at National Institute of Biotechnology and Genetic Engineering (NIBGE) Faisalabad, Pakistan for bacterial isolation. For this purpose, plastic pots of about 1 kg capacity were filled with 1 kg soil. Five to six surface sterilized seed of maize (*Z. mays* L.) were sown in each pot. After 40 days of germination, plants were harvested. Rhizosphere-associated bacteria were isolated by taking 1 g of roots with tightly adhering soil using serial dilution plating technique on LB agar plates (Hameed et al., 2004). The suspension was spread on LB agar plates and incubated at 28 + 2^∘^C till the appearance of bacterial colonies. Individual colonies were picked and streaked on LB plates for further purification. Differences in cell morphology, i.e., cell shape, motility, acid/alkali production, and gram staining were performed by using phase contrast light microscope as described by Vincent (1970). The ability of the isolates to grow in diverse temperature range was carried out by growing isolates in nutrient broth and incubated at different temperatures ranged from 20 to 40^∘^C. Growth was recorded every 24 h up-to 96 h. The ability of the isolates to grow in alkaline or acidic media was tested in nutrient agar plates in which the pH was adjusted from 5.0 to 8.0 and incubated at 30^∘^C for 3 days.

The cell number was calculated from a calibration curve that relates OD values of a series of culture of known cell density. The OD values of cultures containing about ≤0.4 × 10^9^ cell/mL and designated as slow growth (+) ranged from 0.1 to 0.5. The OD values categorized as optimum growth and denoted by (++) ranged from 0.6 to 0.9 while the OD values containing about ≤0.8–2 × cell/mL and designated as maximum growth and is denoted by (+++) ranged ≥1.0 as shown in **Table 2**.

**Table 2:** **Colony and cell morphology of selected bacterial isolates and their temperature and pH tolerance ability**.

Isolate code	Shape	Margins	Color	Cell shape	Gram s’ reaction	Temperature tolerance (^∘^C)				pH tolerance			


						25	30	35	40	5	6	7	8
HJR_1_	Round	Smooth	Off white	Medium rod	+ve	+ + +	+ +	+	+	+	+ + +	+ +	+
HJR_2_	Round	Smooth	Off white	Medium rod	+ve	+	+	+ + +	+	+	+ + +	+ +	+
HJR_3_	Round	Smooth	Off white	Medium rod	+ve	+	+ + +	+ +	+	+ +	+ + +	+ +	+
HJR_4_	Round	Smooth	Glossy white	Small rod	+ve	+	+ +	+ + +	+	+	+ + +	+ +	+ +
HJR_5_	Round	Smooth	Off white	Small rod	+ve	+	+ +	+ + +	+	+ + +	+	+	+
MR_6_	Round	Smooth	white	Medium rod	-ve	+ + +	+	+	+	+	+	+ + +	+ +
HJR_7_	Irregular	wavey	Off white	Medium rod	+ve	+	+	+ + +	+	+	+	+ + +	+
HJR_8_	Round	Smooth	whitish	Long rod	+ve	+	+ + +	+	+	+	+	+ + +	+ +

*Slow growth (+), optimum growth (++), maximum growth (+ + +).*

### Characterization of Bacteria for Plant Growth Promoting Potential

A total of 100 bacterial isolates were screened for their ability to produce indole-3-acetic acid (IAA), phosphate solubilization, and N_2_ fixation. For IAA production, individual bacterial cultures were grown in LB broth supplemented with tryptophan (100 mg/L) as a precursor of IAA at 28 ± 2^∘^C with constant shaking (Okon et al., 1977). After 1 week of growth, IAA was extracted from acidified cell-free supernatant using ethyl acetate and analyzed on high-performance liquid chromatograph equipped with Turbochrom software (Perkin Elmer, USA) and C-18 column at a flow rate of 0.5 ml min^-1^ (Malik et al., 1994). Isolates were categorized into two groups based on their ability to produce IAA *in vitro* as low IAA producers (1–4 μg mL^-1^) and medium IAA producers (5–10 μg mL^-1^) as reported earlier (Khalid et al., 2004). Pure bacterial colonies were inoculated into NFM (Nitrogen Free Malate medium) semisolid medium in vials and incubated at 28 + 2^∘^C for 48 h. Acetylene (10% v/v) was injected to the vials, incubated at room temperature for 16 h and 100 μL of gas sample from each vial was analyzed on a Gas Chromatograph (Thermoquest, Trace G.C, Model K, Rodono, Milan, Italy) equipped with a Porapak Q column and a H_2_ -flame ionization detector (FID).

To determine phosphate solubilization ability, each bacterial culture was spot inoculated on Pikovskaya (1948) agar plates containing tricalcium phosphate as insoluble phosphate source. The plates were incubated at 28 + 2^∘^C for 7–10 days. Appearance of a clear zone around bacterial colonies indicated the P-solubilization ability of the isolate. Total solubilized phosphate was measured by estimating the available phosphorus in the cell-free supernatant by phospho-molybdate blue color method using spectrophotometer (Halder et al., 1990).

### Identification of Potent PGPR based on 16S rRNA Sequencing

Total genomic DNA was extracted by alkaline lysis method (Maniatis et al., 1982). Eubacterial primers fD1 (5^′^-AGA- GTTTGATCCTGGCTCAG-3^′^) and rD1 (5^′^-AAGGAGGTGAT- CCAGCC-3^′^) which correspond to *Escherichia coli* 16S rRNA gene were used for PCR amplification as described by Weisburg et al. (1991). Amplified PCR products were resolved on 1% agarose gel. Purification of amplified products was done by using Purelink^TM^ Quick Gel Extraction Kit (Invitrogen) according to manufacturer protocol. The PCR were sequenced commercially by Macrogen (Korea). The gene sequences were compared with others in the GenBank database using the NCBI BLASTn. Multiple sequence alignments were performed by ClustalX and phylogeny was determined by neighbor-joining method (Saitou and Nie, 1987).Tree topology based on re-samplings of 1000 times of the neighbor joining data set was evaluated by boot strap analysis (Felsenstein, 1985).

### Plant Inoculation Experiment

Influence of various PGPR isolates on growth and N and P concentration on maize (*Z. mays* L.) plant was examined in pots under greenhouse conditions. Eight PGPR strains isolated from the 100 screened isolates (HJR_1_, HJR_2_, HJR_3_, HJR_4_, HJR_5_, MR_6_, HJR_7_, and HJR_8_) along with un-inoculated control and two levels of N and P fertilizer (½toolNP and full NP fertilizer) were selected. The surface sterilized seeds were inoculated by immersion in the PGPR suspension for 1 h. Cleaned earthen pots of 20 cm height and 15 cm depth were used. A combination of about 4 kg soil and 1 kg sand was used for filling in each pot. Four to five air dried surface sterilized seeds were sown in each pot. The experiment was laid down in a completely randomized design with 11 treatments, three replications and total of 33 pots were used in the experiment. Treatments included: (1) HJR_1_+½toolNP (2) HJR_2_+½toolNP (3) HJR_3_+½toolNP (4) HJR_4_+½toolNP (5) HJR_5_+½toolNP (6) MR_6_+½toolNP (7) HJR_7_+½toolNP (8) HJR_8_+½toolNP (9) control (without NP and inoculation; 10) ½toolNP (11) Full NP. Nitrogen and P were applied at the rate of 60 and 45 mg kg^-1^ (full dose) in the form of urea and single super phosphate, respectively. Pots were kept under greenhouse conditions and equally irrigated when needed. The plants were harvested at 30, and 60 days after germination and following measurements were taken. Morphological characteristics and nutrient contents were determined such as shoot and root length, shoot and root dry weight, root surface area (Scanned image analysis program software), leaf surface area (electronic planimeter), N and P contents in shoot and root. The N and P analysis in shoot and root were carried out using the methods described by Winkleman et al. (1990).

### Statistical Analysis

The data were subjected to analysis of variance using statistical program (MSTATC, 1990). The differences among various treatment means were compared using the least significant differences test (LSD) at 5% (*P*≤ 0.05) probability level (Steel and Torri, 1980).

## Results and Discussion

### Isolation and Characterization of PGPR

Among the 100 bacterial isolates from the twenty maize growing sites, eight named as HJR_1_, HJR_2_, HJR_3_, HJR_4_, HJR_5_, MR_6_, HJR_7_, and HJR_8_ were selected based on their ability to produce phytohormone IAA, solubilize insoluble phosphate, or fix N_2_. These bacterial isolates were characterized based on their morphological features such as shape, margins, color, and pigmentation. All eight isolates were fast growing, having round to irregular colony shape with raised elevation and smooth surface. No pigmentation was produced by any of the tested isolate on LB medium (**Table 2**). Isolates HJR_4_ and HJR_5_ were small rod; HJR_1_, HJR_2_, HJR_3_, MR_6_, and HJR_7_ were medium rods; and HJR_8_ showed long rods. Except MR_6_, all isolates were Gram positive. The isolates were grown in ranges of temperature and pH to examine their tolerance to pH and temperature extremes. The results indicated that almost all isolates were able to grow in temperatures ranging between 25 and 40^∘^C, and pH ranging between 5 and 8, with an optimum temperature of 35^∘^C and pH of 7.0 (**Table 2**). Our results are similar to those reported for PGPR isolated from apple rhizosphere in Himachal Pradesh, India (Mehta et al., 2014). The apple rhizosphere isolates reported by Mehta et al. (2014) had an optimum growth temperature of 30^∘^C and pH of 7.0 with similar phenotypic characteristics as observed in our study.

### Characterization of Bacterial Isolates

**FIGURE 1:**
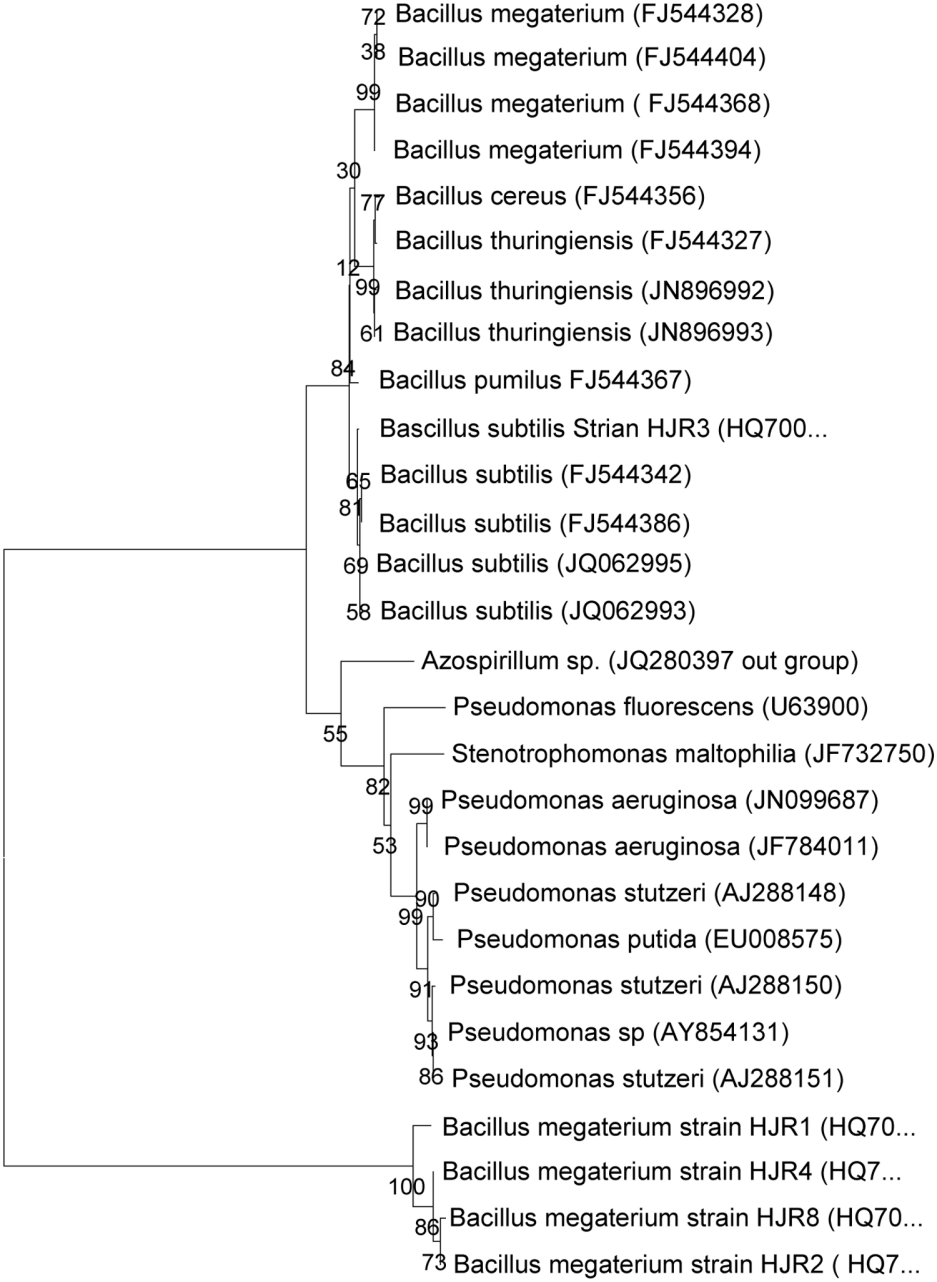
Phylogenetic tree of 16S rRNA gene sequences showing the relationships among the isolates isolated from the soils of Himalayan region of Hajira Rawalakot, Azad Jammu and Kashmir (AJK), Pakistan and the related genera. The data of type strains of related species were from GenBank database (the accession numbers are given in parentheses).

The sequence analysis of 1.5 kb fragment of 16S rRNA gene of all eight bacterial isolates was analyzed by nucleotide Blast analysis. The sequence of isolates HJR_1_ and HJR_3_ showed 99% similarity with *Bacillus subtilis* and were submitted to GenBank under accession number HQ700330 and HQ700332, respectively. Isolates HJR_2_, HJR_4_, and HJR_8_ had 99% sequence match with that of *Bacillus megaterium*. These isolates were submitted to GenBank under accession number HQ700331, HQ700333, and HQ700334, respectively. Isolate MR_6_ showed similarity with genus *Pseudomonas* and was identified as *Pseudomonas stutzeri*. These sequences were aligned with sequence of some PGPR of different genera and species within genera from the GenBank database. The phylogenetic tree of these strains constructed by using their 16S rRNA sequences (**Figure 1**) showed that the selected isolates were members of genus *Pseudomonas* and *Bacillus.*

*Bacillus* and *Pseudomonas* are the most commonly reported genera and represent the dominant isolates in many plant studies (Hallmann and Berg, 2006). The number of isolates belonging to the genus *Bacillus* was higher in this study similar to those reported earlier explaining that *Bacillus* spp. are dominant in root adhering soil (Laguerre et al., 1994). Studies on the diversity of root-associated bacteria in maize carried out in different geographical regions, revealed extensive colonization by *Bacillus* sp. during the active growth stage of the plants (Lambert et al., 1987; Lalande et al., 1989). Both these studies also reported a substantial or rather predominant fraction of *Pseudomonas* present in the isolation pool (reported in Grönemeyer et al., 2012).

### Characterization for Plant Growth Promoting Traits

The isolates associated with the roots of maize crop were tested for features known to contribute to plant growth promotion or biocontrol (**Table 3**).

**Table 3:** **Plant growth promoting potential of different bacterial isolates under *in vitro* condition**.

Isolate code	Identification based on 16S rRNA sequencing	Phosphate solubilization (mg mL^-1^)	Decrease in pH of medium	IAA production (μg mL^-1^)	Acetylene reduction assay
HJR_1_	*Bacillus subtilis*	25.7	4.2	0.9	+
HJR_2_	*B. megaterium*	27.4	4.8	4.8	+
HJR_3_	*B. subtilis*	35.6	3.5	2.6	+
HJR_4_	*B. megaterium*	22.05	4.5	1.4	–
HJR_5_	*B. megaterium*	23.15	4.7	1.9	+
MR_6_	*Pseudomonas stutzeri*	19.2	4.9	2.9	–
HJR_7_	*Bacillus* sp.	28.9	4.2	5	–
HJR_8_	*B. megaterium*	25.7	4.4	5.3	–

### Production of Indole-3-Acetic Acid

All *Bacillus* and *Pseudomonas* spp. produced IAA *in vitro* in tryptophan supplemented LB medium. Isolates HJR_2_, HJR_7_, and HJR_8_ were medium producers of IAA (4.8–5.3 μg ml^-1^) while HJR_1_, HJR_4_, HJR_5_, HJR_6_ designated as weak producers (0.9–2.9 μg ml^-1^; **Table 3**). The amount of IAA produced by these isolates was substantially lower than that reported earlier from other regions (Fischer et al., 2007; Tahir et al., 2013) but similar to that reported under similar environmental conditions from this region (Abbasi et al., 2011; Zhang et al., 2012). However, it has been reported that the amount of indole compounds produced *in vitro* depends on the particular bacterial species, strain, or the conditions of the culture such as oxygenation and pH (Radwan et al., 2002). The variation among PGPRs to produce IAA found in the present study had also been reported earlier (Zahir et al., 2000; Abbasi et al., 2011). This variation is attributed to the various biosynthetic pathways, location of the genes involved, regulatory sequences, and the presence of enzymes to convert active free IAA into conjugated forms (Patten and Galick, 1996). A high level of IAA production was recorded in different strains of bacteria with the members of the genera *Pseudomonas* spp., *Bacillus* spp., *Rhizobium,* and *Mesorhizobium* spp. by other workers (Ahmad et al., 2008; Verma et al., 2012, 2013).

### Solubilization of Inorganic Phosphates

The results showed that all isolates had P solubilization potential ranging between 19.2 and 35.6 μg mL^-1^ (**Table 3**). The highest P solubilization was measured for bacterial isolate HJR_3_ (35.6 μg mL^-1^) fallowed by HJR_7_ (28.9 μg mL^-1^) and HJR_2_ (27.4 μg mL^-1^). The solubilization of TCP by different isolates was accompanied by a significant drop in pH (4.9–3.5) from an initial pH of 7.0. The maximum drop in culture pH of 3.5 was associated with isolate HJR_3_. P-solubilization activity was associated with the release of organic acids and a drop in pH. Pikovskaya’s medium indicated the efficacy of tested isolates to solubilize the unavailable P (Mehta et al., 2014). It has been reported that P solubilization is mainly due to the production of microbial metabolites including organic acids which decrease the pH of the culture media (Puente et al., 2004; Sahin et al., 2004).

The ability of PGPR strains to solubilize insoluble P and convert it to plant available form is an important characteristic under conditions where P is a limiting factor for crop production. In general, out of the 100 isolates tested, only eight were able to show P solubilization. Such low percentage of isolates that show P solubilization ability is not unique to our study as other studies also show limited numbers. Two separate studies in pearl millet and rice paddy field indicated that only 5% of the 207 total tested strains in of the studies and 23.5% in the other possessed P solubilizing ability (Hameeda et al., 2006; Rashedui et al., 2010). The presence of P-solubilizing microbial population in soils may be considered a positive indicator of utilizing the microbes as biofertilizers for crop production and beneficial for sustainable agriculture.

Based on the acetylene reduction assay, isolates HJR_1_, HJR_2_, HJR_3_, and HJR_5_ showed the ability to fix N_2_. The remaining isolates did not show any potential for N_2_ fixation (**Table 3**). The presence of N_2_ fixing bacteria in soil and its isolation and conversion into PGPR biofertilizer is an important strategy reducing the use of expensive chemical fertilizers especially in nutrient poor and degraded soils. Biological N_2_ fixation provides a major source of N for plants as part of environmental friendly agricultural practices (Cakmakci et al., 2006).

### Plant Growth Promotion

Co-inoculation of these isolates along-with 50% reduced fertilizer dose (½toolNP) was tested against un-inoculated/unfertilized control and with two levels of NP fertilizer (½toolNP and full NP fertilizer) to evaluate their potential as PGPR in maize (**Table 4**). Results indicated that all isolates significantly (*P*≤ 0.05) increased the growth of maize compared to the control and in some cases to that recorded under ½toolNP treatment. The shoot length, root length, shoot fresh weight, and root fresh weight were highest in HR_3_+½toolNP and full NP treatments. Root dry weight and leaf area were significantly (*P*≤ 0.05) higher in HR_3_+½toolNP compared to the remaining treatments including full NP. Root area was maximum in HR_8_+½toolNP. Most of the isolates when combined with ½toolNP showed significantly higher growth characteristics compared to the treatments supplemented with ½toolNP. The difference in growth between isolates+½toolNP and ½toolNP treatments was due to the addition of PGPR isolates. The results indicated that the growth traits recorded under HJR_3_+½toolNP treatment were significantly (*P*≤ 0.05) higher than those recorded under other isolates, ½toolNP and control treatments but at par with those recoded under full NP fertilizer treatment showing the dominating and promising effect of isolate HJR_3_ over the remaining isolates (**Table 4**).

**Table 4:** **Effect of inoculation with plant growth promoting rhizobacteria on the growth of maize (*Zea mays* L.) grown in pots under greenhouse conditions**.

Treatments	Shoot length (cm)	Root length (cm)	Shoot fresh wt. (g plant^-1^)	Root fresh wt. (g plant^-1^)	Shoot dry wt. (g plant^-1^)	Root dry wt. (g plant^-1^)	Root area (cm^2^)	Leaf area (cm^2^)
HJR_1_+½toolNP	56.7h	35.6d	20.4gh	19.2e	10.27c	3.6b	228.1e	7.85ab
HJR_2_+½toolNP	53.4i	19.1h	27.7d	19.3e	7.92de	2.7c	216.4f	4.83d
HJR_3_+½toolNP	95.2a	44.0a	38.0a	36.1a	18.20a	5.6a	350.5b	8.75a
HJR_4_+½toolNP	74.1e	23.6g	21.7fg	17.1f	8.39d	2.7c	270.0c	5.33d
HJR_5_+½toolNP	78.3d	27.5f	23.0ef	19.0e	6.43f	2.0cd	235.4d	4.60de
MR_6_+½toolNP	61.8g	33.6e	28.7c	17.0f	6.67ef	1.6de	206.4g	3.84e
HJR_7_+½toolNP	88.0c	41.3bc	34.6b	31.5b	13.40b	2.1cd	221.7f	6.31c
HJR8+½toolNP	86.6c	40.0c	30.0c	24.6d	8.79d	6.0a	406.8a	4.73de
Zero Control	47.9j	16.5i	14.6 j	11.8i	4.13g	1.1e	109.3j	2.20f
½toolNP control	65.8df	28.0cf	22.5d	19.0e	5.50f	1.8de	182.0h	2.04e
Full NP control	93.8a	42.7a	36.8a	34.9a	14.44b	6.3a	136.4j	7.63b
LSD (*P ≤ 0.05*)	2.60	1.97	1.75	1.81	1.31	0.87	5.88	0.94

*Samplings for the above characteristics were taken twice, i.e., 30 and 60 days after germination and the values presented are the average of the two samplings.*

Plant growth promotion in response to PGPRs applied alone or with N or P fertilizers has been reported recently for different crops under different ecological and environmental conditions (Lee et al., 2012; Krey et al., 2013; Verma et al., 2013; Lavakush et al., 2014; Mehta et al., 2014). The PGPRs may promote the plant growth either by using their own metabolism (solubilizing phosphates, producing hormones, or fixing nitrogen) or directly affecting the plant metabolism (increasing the uptake of water and minerals), enhancing root development, increasing the enzymatic activity of the plant or “helping” other beneficial microorganisms to enhance their action on the plants, or by suppressing plant pathogens (Pérez-Montaño et al., 2014). The PGPRs tested in this study possessed multiple plant growth promoting traits including P-solubilization, IAA production, and N_2_ fixation.

### N and P Concentration in Maize Plants

**FIGURE 2:**
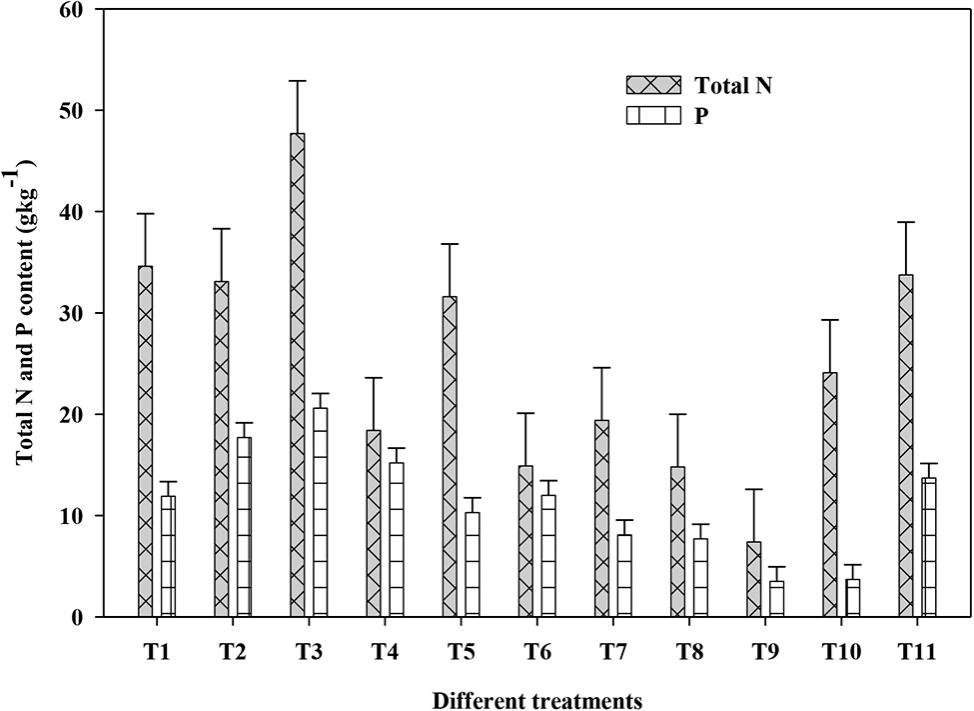
Effect of inoculation with *Bacillus* and *Pseudomonas* on the shoot and root N and P contents of maize plant grown under greenhouse condition after 60 days. Treatments included T_1_, HJR_1_+½toolNP; T_2_, HJR_2_+½toolNP; _T3_, HR_3_+½toolNP; T_4_, HJR_4_+½toolNP, T_5_, HJR_5_+½toolNP; T_6_, MR_6_+½toolNP; T_7_, HJR_7_+½toolNP; T_8_, HJR8+½toolNP; T_9_, Control; T_10_, ½toolNP; T_11_, Full NP. The vertical lines on each bar represent the standard error of mean (SEM, *n* = 3).

The N and P contents of both shoot and root of plants in response to PGPR isolates, NP fertilizer and control treatments is presented in **Figure 2**. The N and P contents in both shoot and root showed similar trend in response to different treatments; hence, the N and P contents is presented here as a total of the shoot and root. The total N in plants received the control treatment was 7.4 g kg^-1^ compared with 14.8–47.7 g kg^-1^ for plants those received the PGPRs and NP fertilizers, showing a 2–6 fold increase. Among the different amendments, co-inoculation with isolate HJR_3_+½toolNP displayed superiority over the remaining treatments including full NP treatment. The highest total N content (in shoot+root) of 47.7 g kg^-1^ was recorded in plants grown under HJR_3_+½toolNP followed by 34.6, 33.8, and 33.1 g kg^-1^ N under HJR_1_+½toolNP, full NP and HJR_2_+½toolNP treatments, respectively. The relative increase in total N contents under HJR_3_+½toolNP over HJR_1_+½toolNP, full NP and HJR_2_+½toolNP was 38, 41, and 44%, respectively. The N contents observed in HJR_3_+½toolNP was almost double to those recorded under ½toolNP treatment showing that the additional N in plant was eventually because of isolate HJR_3_.

Total P contents both in shoot and root also showed similar trend as that of total N (**Figure 2**). The minimum total P content of 3.5 and 3.7 g kg^-1^ was found under the control and ½toolNP fertilizer treatments. Application of different PGPRs with ½toolNP significantly increased plant P contents to between 7.7 and 20.6 g kg^-1^ showing a 2–6 fold increase due to inoculation with PGPRs. The highest P content of 20.6 g kg^-1^ was recorded under HJR_3_+½toolNP treatment followed by 17.7 g kg^-1^ P under HJR_2_+½toolNP. The full NP fertilizer treatment had 13.7 g kg^-1^ total P content. The relative increase in P contents in plants grown under HJR_3_+½toolNP and HJR_2_+½toolNP over full NP was 50 and 29%, respectively.

These results show the efficient transfer of N and P to plants by the PGPR strains as reported earlier rice (Islam et al., 2009), wheat (Abbasi et al., 2011), common bean (*Phaseolus vulgaris* L.; Tajini et al., 2012), and chickpea (*Cicer arietinum* L.; Verma et al., 2013). This increase in NP concentration in plant tissues is associated with N_2_ fixation and P solubilization potential of applied PGPRs. The highest N concentration was recorded in plants supplemented with isolates HJR_1_, HJR_2_, HJR_3_, and HJR_5_. All of these four isolates showed nitrogenase activity (N_2_ fixation; **Table 3**) signifying a close association between plant N concentration and N_2_ fixation. In our previous study, the N content in wheat shoot under control was 1.2% that significantly increased to 1.7–2.43% by the application of different PGPR strains (Abbasi et al., 2011).

The increase in plant P concentration in response to PGPRs is attributed to the fact that PGPRs have the ability to solubilize insoluble phosphates, making it available for plant uptake through different mechanisms such as acidification, chelation, and ion-exchange reactions (Coutinho et al., 2012). The results presented in **Table 3** indicate a substantial potential of applied strains to solubilize P and increase P concentration in plants. The increased concentration of N and P in plants supplemented with PGPRs suggest that a positive interaction exists between root colonization, N and P concentration and growth promotion. Further, this study suggests that plant N derived from N_2_-fixation and P concentration as a result of P-solubilization by phosphate solubilizing microorganism is substantially enhanced above those of uninoculated control plants.

The significant increase in growth and NP level both in shoot and root upon isolates application is a clear indication that bacterial isolates are able to provide better nutrient flux to the plant and result in increased plant biomass. The increase in root length and mass due to the applied isolates may also be a factor that contributes to the increase in N and P concentration in plant shoot and root.

## Conclusion

This study indicates the presence of some efficient and effective species of bacteria in the soils of mountainous region of Rawalakot, AJK, Pakistan. Our results demonstrate that efficient N_2_ fixing and P solubilizing isolates are present among natural population of rhizobacteria. These characteristics are important growth promoting traits for plants growing in the region under continuous threat of soil erosion and soil degradation. The combination of *Bacillus* spp. with ½toolNP fertilizer resulted in plant growth equivalent to full NP fertilizer treatment while the N and P concentration in plant biomass in this combined treatment was even higher than that recorded under full NP treatment. The effectiveness of PGPR isolates with NP fertilizers clearly indicates that the chemical fertilizers rate or dose could be reduced through combination of PGPR isolates with fertilizers that may be an eco-friendly and cost effective management strategy. These native isolates may be used as efficient bio-inoculants for integrated nutrient management in the uplands soils facing severe threat of erosion and degradation. Therefore, these isolates might have potential in future field applications as plant growth promoters. The future studies should be focused on the functional characterization of PGPR for practical applications in the field.

## Conflict of Interest Statement

The authors declare that the research was conducted in the absence of any commercial or financial relationships that could be construed as a potential conflict of interest.
